# Comparison of knowledge, attitude, and practices of animal and human brucellosis between nomadic pastoralists and non-pastoralists in Kenya

**DOI:** 10.1186/s12889-020-8362-0

**Published:** 2020-02-24

**Authors:** M. Kariuki Njenga, Eric Ogolla, Samuel Mwangi Thumbi, Isaac Ngere, Sylvia Omulo, Mathew Muturi, Doris Marwanga, Austine Bitek, Bernard Bett, Marc-Alain Widdowson, Peninah Munyua, Eric Mogaka Osoro

**Affiliations:** 1 Washington State University Global Health Program, Washington State University, Nairobi, Kenya; 2 Jaramogi Oginga Odinga University of Science and Technology, Bondo, Kenya; 3 Kenya Ministry of Agriculture and Fisheries, Nairobi, Kenya; 4 Center for Global Health Research, Kenya Medical Research Institute, Nairobi, Kenya; 5 United Nations Food and Agriculture Organization-Kenya Office, Nairobi, Kenya; 6 International Livestock Research Institute, Nairobi, Kenya; 7 Division of Global Health Protection, Centers for Disease Control and Prevention-Kenya, Nairobi, Kenya

**Keywords:** Brucellosis, Knowledge, Risky practices, Kenya

## Abstract

**Background:**

The seroprevalence of brucellosis among nomadic pastoralists and their livestock in arid lands is reported to be over10-fold higher than non-pastoralists farmers and their livestock in Kenya. Here, we compared the seroprevalence of nomadic pastoralists and mixed farming with their knowledge of the disease and high-risk practices associated with brucellosis infection.

**Methods:**

Across-sectional study was conducted in two counties - Kiambu County where farmers primarily practice smallholder livestock production and crop farming, and Marsabit County where farmers practice nomadic pastoral livestock production. Stratified random sampling was applied, in which sublocations were initially selected based on predominant livestock production system, before selecting households using randomly generated geographical coordinates. In each household, up to three persons aged 5 years and above were randomly selected, consented, and tested for *Brucella spp* IgG antibodies. A structured questionnaire was administered to the household head and selected individuals on disease knowledge and risky practices among the pastoralists and mixed farmers compared. Multivariable mixed effects logistic regression model was used to assess independent practices associated with human *Brucella spp*. IgG seropositivity.

**Results:**

While the majority (74%) of pastoralist households had little to no formal education when compared to mixed (8%), over 70% of all households (pastoralists and mixed farmers) had heard of brucellosis and mentioned its clinical presentation in humans. However, fewer than 30% of all participants (pastoralists and mixed farmers) knew how brucellosis is transmitted between animals and humans or how its transmission can be prevented. Despite their comparable knowledge, significantly more seropositive pastoralists compared to mixed farmers engaged in risky practices including consuming unboiled milk (79.5% vs 1.7%, *p* < 0.001) and raw blood (28.3% vs 0.4%, *p* < 0.001), assisting in animal birth (43.0% vs 9.3%, *p* < 0.001), and handling raw hides (30.6% vs 5.5%, *p* < 0.001).,

**Conclusion:**

Nomadic pastoralists are more likely to engage in risky practices that promote *Brucella* Infection, probably because of their occupation and culture, despite having significant knowledge of the disease.

## Background

Brucellosis is a globally widespread zoonotic disease that causes substantial morbidity in both livestock and human populations, particularly in Latin America, Middle East, and Africa where it is endemic [1]. Of the six species of the bacteria, *Brucella abortus* and *Brucella melitensis* are the predominant species associated with human disease. These *Brucella* species are transmitted from infected animals primarily through inhalation of the bacteria, consumption of contaminated unpasteurized dairy products, and direct contact with infected animal fluids and tissues [1, 2]. While the disease is rarely fatal, human brucellosis is a chronic debilitating and disabling disease that is often difficult to diagnose and requires long and expensive antibiotic treatment, which may not always be successful [3, 4]. Among livestock (cattle, sheep, goats and camels), *B. abortus* and *B. melitensis* are spread through contact with infected birthing tissues and fluids via ingestion or direct contact with mucous membranes and sexually [1, 2]. Brucellosis infection in livestock is often chronic, leads to abortions and infertility and is associated with major economic losses associated with reduced productivity in animals, and trade restrictions [5].

In endemic countries, the seroprevalence of brucellosis in livestock varies from < 1 to 30% [6–10]. In these regions, human incidence of the disease ranges widely, with areas such as Africa and Middle East reporting between 50 and 250 cases per 100,000 [3]. Most data show human seroprevalence of brucellosis is positively correlated with livestock seroprevalence, emphasizing the role of livestock as the source of human infections [3]. Public education in combination with livestock vaccination has been shown to reduce disease incidence in humans and animal populations through adoption of risk reduction practices [11]. Interestingly, many studies show significant knowledge of brucellosis among rural and urban populations in developing countries; with between 40 and 100% of populations reporting awareness of the disease and its clinical presentations [8, 12–14]. However, few studies have been carried out among nomadic pastoralist communities residing in underdeveloped remote and arid areas and deriving livelihood primarily from rearing livestock- where infection risk is likely elevated [13, 15].

In Africa, over 100 million nomadic pastoralists, living in the underserved arid and semi-arid lands of the continents own more than 30% of all livestock and 50% of small ruminants, supplying 60% of beef and 40% of sheep and goat meat in the countries where they inhabit (FAO, 2012). In 2013, a study in Kenya reported a 12-fold higher seroprevalence of brucellosis among nomadic pastoralist livestock herds in northern Kenya compared to mixed farmers (livestock and crops) in central Kenya, and 14-fold higher prevalence in pastoralist households (humans) when compared to mixed farmers [10]. Similarly, the livestock prevalence (cattle, sheep, goats and camels) was 11-fold higher and human prevalence 19-fold higher among pastoralists when compared to mixed farmers. A breakdown of the seroprevalence among livestock species gave a range of 11–16% prevalence in pastoral livestock, compared to 0.8–2.4% in livestock reared in small-scale production systems [10].

Here, we compared the knowledge of brucellosis and risky practices of infection among seropositive households in two communities; nomadic pastoralists living in the remote, underdeveloped and arid northern region, and mixed farmers living in a developed, high potential, agro-ecological region of Kenya.

## Methods

### Study design and sample size determination

**Fig. 1:**
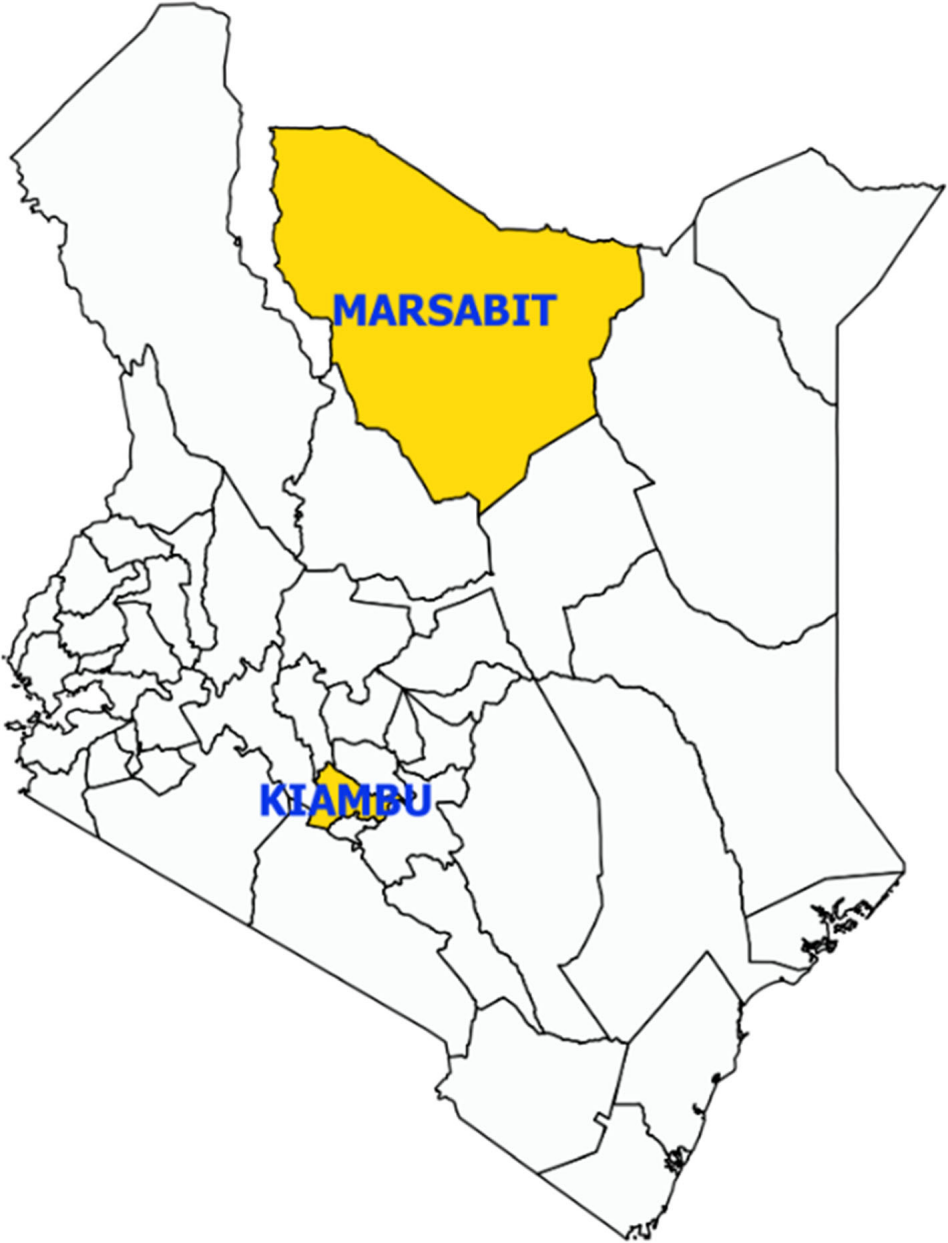
Map of Kenya showing the location of Marsabit and Kiambu counties. Map created in QGIS

A cross sectional study compared knowledge and practices related to brucellosis between participants from the predominantly nomadic pastoralists of Marsabit County and that of mixed farmers of Kiambu County (Fig. 1). Kiambu County neighbors Nairobi, the capital city of Kenya, and is located in a high potential agro-ecological zone with farmers practicing smallholder livestock production (keeping primarily cattle, sheep and goats) and crop farming. Marsabit County is located in the northern arid agro-ecological zone of the country and farmers practice nomadic pastoral livestock production mainly, keeping cattle, sheep, goats and camels. The estimated livestock population in Marsabit County is 2,731,407, of which 42% are goats, 35% are sheep, 16% are cattle and 7% are camels; whereas Kiambu County has a livestock population of 1,832,045 of which 39% are sheep, 38% are goats, 22% cattle and < 1% camels.

Kiambu County has good physical infrastructure with 35% of the roads tarmacked or on gravel, accessible medical and veterinary services, and is densely populated with over 630 persons per square kilometer, inhabited by a community with high literacy levels, more than 45% of them deriving livelihood from the Capital City of Nairobi [16]. In contrast, Marsabit County has only one major road with most areas inaccessible for medical or veterinary services and is sparsely populated with 4 persons per square kilometer, inhabited by a poor, nomadic pastoralist communities that derive their livelihood from rearing livestock, including cattle sheep, goats, and camels [17].

This study was part of a larger study on seroprevalence of and risk factors for brucellosis infection in humans and livestock in Kenya whose findings were published previously [10]. The sample size was calculated based on an estimated *Brucella* spp. seroprevalence of 5% in Kiambu County and 50% in Marsabit County, with an error margin of 2 and 5%, respectively, at 95% confidence level. A design effect of two and a factor of 10% were applied to account for clustering and non-response respectively, giving a minimum sample size of 730 individuals for Marsabit and 866 individuals for Kiambu counties.

### Household selection and sampling

The study applied stratified random sampling to identify study households in each county [10]. In the first stage, sub-locations were stratified by predominant livestock production system and 10% of sub-locations were randomly selected from each stratum in each county. This resulted in 21 sub-locations in Kiambu County and 10 in Marsabit County. In the second stage, the number of households to be visited in each sub-location were determined proportionate to the total human population and assuming an enrolment of three persons per household. In order to identify households to recruit into the study, random geographical coordinates were generated using ArcGIS corresponding to number of households for each sublocation. The selected household coordinates were loaded into a global positioning system device used by each study team. When the coordinates did not correspond to a household, the nearest household was visited. In each household, up to three persons aged 5 years and above were randomly selected, consented/assented in line with the ethical approval, and a structured questionnaire, loaded on to a smartphone, administered to each participant and the household head. Nomadic pastoralists were defined as households whose livelihood was based primarily on domesticated livestock production and involved seasonal movement of dwelling. Mixed farming were households whose livelihoods depended on both livestock rearing and crop farming.

### Data and sample collection and laboratory testing

We used an electronic interviewer administered structured questionnaire with standardized questions and scheme to collect data from household respondents on knowledge and practices that may be associated with increased risk of infection with *Brucella spp.* The questionnaire was pretested, and interviewers trained before data collection. The data collected included knowledge of human and animal brucellosis including transmission, symptoms and modes of prevention. The study also collected data on practices at individual level including drinking of unboiled milk, assisting in animal birthing, drinking raw blood, working with raw hides and skins. Weekly frequencies on selected variables were done to check on data quality. A blood sample was collected from all eligible persons and animals as previously reported [10]. After processing for sera, the specimens were tested for presence of anti-*Brucella* spp. IgG antibodies using IBL-America IgG enzyme-linked immunosorbent assay (ELISA) and Svanova Biotech AB ELISA kits for human and animal samples respectively as we previously reported [10].

### Data analysis

Data were analysed using R statistical software, version 3.5.1 [18]. Categorical variables were presented as percentages and their associations assessed by Chi-square test while continuous variables were tested using the t-test. Knowledge on human and animal brucellosis by household heads was presented by production system practiced by the household (nomadic pastoralism vs mixed farming).

The prevalence of practices among participants from households practicing nomadic pastoralism or mixed farming was compared. We conducted a multivariable mixed effects logistic regression model with human *Brucella spp*. IgG seropositivity as the outcome variable and included the practices, sex, age, and education level as predictor variables. Household was included in the model as a random effect to account for possible clustering. *P*-values < 0.05 were considered significant. Missing values were excluded from the analysis and a goodness-of-fit test was conducted on the model using Hosmer-Lemeshow test (*p* > 0.05).

### Ethical approval

The study received ethical approval by the Kenya Medical Research Institute Scientific Ethical Review Committee (No. 2193) and Centers for Disease Control and Prevention Institutional Review Board. Project approval was also obtained from the Kenya Ministry of Health, and the Ministry of Agriculture Livestock and Fisheries.

## Results

### Enrolled households and demographic data for household heads

A total of 787 households were enrolled, of which 510 (65%) were from Kiambu and 277 (35%) from Marsabit. There were significantly more female household head respondents (57.3%) in Kiambu than Marsabit County (49.7%) (*p* < 0.001). The average age of household heads respondents was 36.7 years (SD 19.2, range = 5–96) in Kiambu, and 34.4 years (SD 19.9, range = 5–90) in Marsabit.

Of 787 enrolled households, 47% (*n* = 371) reported practicing mixed farming, 26% (*n* = 204) nomadic pastoralism, 4% (*n* = 35) peri-urban livestock farming with no crops or movement, 21% (*n* = 169) did not own any livestock and 1% (*n* = 8) had missing data. Nearly all the nomadic pastoralists (96.1%) were from Marsabit County while 92.2% of households practicing mixed farming were from Kiambu County (Table 1). Our subsequent analyses on knowledge and practices were based on 575 (73%) households, which either practiced nomadic pastoralism or mixed farming.

**Table 1:** Knowledge of human brucellosis among household heads from nomadic pastoralists and mixed farming households, 2012–2013

Variable	Category	Total	Nomadic Pastoralists	Mixed Farmers	*P*-value
		n (%)	n (%)	n (%)	
Total households		575 (100.0)	204 (35.5)	371 (64.5)	
County	Kiambu	350 (60.9)	8 (3.92)	342 (92.2)	< 0.001
	Marsabit	225 (39.1)	196 (96.1)	29 (7.82)	
Heard of brucellosis^a^	Yes	479 (83.6)	171 (83.8)	308 (83.5)	0.874
	No	89 (15.5)	32 (15.7)	57 (15.4)	
Are humans affected by brucellosis^a^	Yes	431 (89.0)	148 (86.0)	283 (90.7)	0.073
	No	11 (2.3)	7 (4.07)	4 (1.28)	
	Don’t know	42 (8.7)	17 (9.9)	25 (8.01)	
How do humans get brucellosis?	Eating uncooked/ undercooked meat from an infected animal	90 (15.7)	11 (5.4)	79 (21.4)	< 0.001
	Milking	59 (10.3)	6 (3.0)	53 (14.4)	< 0.001
	Drinking/eating raw dairy products	26 (4.5)	3 (1.5)	23 (6.2)	0.016
	Contact with aborted animal fetus	13 (2.3)	4 (2.0)	9 (2.4)	0.76
	Herding	10 (1.75)	0 (0.0)	10 (2.7)	0.017
	Slaughtering animals	4 (0.7)	0 (0.0)	4 (1.1)	0.304
	Don’t know	146 (25.5)	68 (33.3)	78 (21.1)	0.003
Signs and symptoms of brucellosis	Chills	437 (76.3)	83 (40.7)	354 (95.9)	< 0.001
	Lack of appetite	195 (34.0)	163 (79.9)	32 (8.67)	< 0.001
	Joint pains	102 (17.8)	94 (46.1)	8 (2.2)	< 0.001
	Fatigue	76 (13.3)	74 (36.3)	2 (0.5)	< 0.001
How is brucellosis prevented?	Boiling milk	104 (18.2)	8 (3.9)	96 (26.0)	< 0.001
	Medication	54 (9.4)	26 (12.7)	28 (7.6)	0.058
	Vaccination	15 (2.6)	0 (0.0)	15 (4.1)	0.01
	Other methods	26 (4.5)	5 (2.5)	21 (5.7)	0.115

^a^Variable has some missing data

### Knowledge of brucellosis disease among household heads

Overall, about 84% of the household heads had heard about brucellosis and about 90% knew it affected humans from among households practicing nomadic pastoralism or mixed farming. The majority (76%) of the respondents reported knowledge of chills as a symptom with about one-third reporting at least one prevention method of or transmission method of human brucellosis (Table 1).

On knowledge of disease, 83.8% (*n* = 204) of nomadic pastoralists and 83.5% (*n* = 371) of mixed farmers had heard of brucellosis. Less than half of the participants from each of the two groups (31.0% among nomadic pastoralists and 39.9% in mixed farmers, *p* < 0.001) knew that the disease affected animals, and less than 20% could list at least one clinical sign in animals including abortion, swollen joints or reduced milk production. On disease transmission, less than 5% of participants from the two groups mentioned consuming raw dairy products or contact with aborted fetuses, as mechanisms of animal-to-human transmission, whereas less than 30% of participants (24.5% in nomadic pastoralists; 25.5% in mixed farmers) mentioned contamination with pastures or contact with wildlife as mechanisms of livestock transmission. Similarly, less than 30% of participants from the two groups knew how to prevent the disease in humans or animals including measures such as boiling milk (3.9% among nomadic pastoralists vs 26.0% among mixed farmers, *p* < 0.001) (Tables 1 and 2).

**Table 2:** Knowledge of animal brucellosis among household heads from nomadic pastoralists and mixed farming households, 2012–2013

Variable	Category	Total	Nomadic Pastoralists	Mixed Farmers	*P*-value
		n (%)	n (%)	n (%)	
Total households		575 (100.0)	204 (35.5)	371 (64.5)	
Are animals affected?^a^	Yes	176 (36.7)	53 (31.0)	123 (39.9)	< 0.001
	No	118 (24.6)	61 (35.7)	57 (18.5)	
	Don’t Know	185 (38.6)	57 (33.3)	128 (41.6)	
Which animals are affected?	Cattle	164 (28.6)	33 (16.2)	131 (35.5)	< 0.001
	Goats	116 (20.2)	60 (29.4)	56 (15.2)	< 0.001
	Sheep	74 (12.9)	31 (15.2)	43 (11.7)	0.251
	Camels	26 (4.5)	19 (9.3)	7 (1.9)	< 0.001
How brucellosis spread among animals?	Ingestion of contaminated pasture	3 (0.5)	52 (25.5)	91 (24.5)	0.226
	Sexually	3 (0.5)	1 (0.5)	11 (3.0)	0.064
	Contact with wild animals	1 (0.2)	3 (1.5)	4 (1.1)	0.697
	Others	314 (54.8)	91 (44.6)	147 (39.8)	0.307
Signs and symptoms of brucellosis?	Abortion	53 (9.3)	38 (18.6)	15 (4.1)	< 0.001
	Swollen joints	33 (5.8)	30 (14.7)	3 (0.8)	< 0.001
	Reduced milk production	23 (4.0)	1 (0.49)	22 (5.96)	0.003
	Swollen testes	3 (0.5)	0 (0.0)	3 (0.8)	0.556
	Infertility	3 (0.5)	1 (0.5)	2 (0.5)	1
	Retained placenta	1 (0.2)	1 (0.5)	0 (0.0)	0.356
	Don’t know	314 (58.8)	76 (37.3)	238 (64.5)	< 0.001
How can brucellosis be prevented?	Drug treatment	70 (12.2)	33 (16.2)	37 (10.0)	0.043
	Vaccination	57 (10.0)	13 (6.4)	44 (11.9)	0.048
	Slaughter	2 (0.4)	2 (1.0)	0 (0.0)	0.126
	Don’t know	287 (50.1)	95 (46.6)	192 (52.0)	0.244

^a^Variable has some missing data

### Practices associated with *Brucella* spp. IgG seropositivity among mixed farmers and nomadic pastoralists

From the 787 enrolled households, 1255 participants were recruited from Kiambu County (an average of 2.5 participant/household) and 765 from Marsabit County (an average of 2.8 participants per household). For this analysis, 562 participants from households practicing nomadic pastoralism and 982 participants from mixed farming households were included. A majority (74.0%) of participants from nomadic pastoralist households had no formal education with only 5.1% completing secondary education or higher. In contrast, only 7.8% of participants from mixed farming household had no formal education with 47.5% completing secondary education or higher (Table 3).

**Table 3:** Comparison of demographic characteristics and practices that promote brucellosis infection between nomadic pastoralists and mixed farmers, 2012–2013

Variable	Category	Nomadic Pastoralists^a^ (*N* = 562)	Mixed Farmers^a^ (*N* = 982)	*P*-value
		n (%)	n (%)	
Sex	Female	277 (49.3)	546 (55.6)	0.019
	Male	285 (50.7)	436 (44.4)	
Age, years	Mean (SD)	34.4 (19.7)	37.6 (20.3)	0.003
Education Level completed^b^	No Formal Education	412 (74.0)	76 (7.8)	< 0.001
	Primary	117 (21.0)	438 (44.7)	
	Secondary	21 (3.8)	354 (36.2)	
	Post secondary	7 (1.3)	111 (11.3)	
Drink Unboiled Milk^b^	Yes	418 (79.5)	16 (1.7)	< 0.001
	No	108 (20.5)	946 (98.3)	
Drink Raw Blood	Yes	159 (28.3)	4 (0.4)	< 0.001
	No	403 (71.7)	978 (99.6)	
Assist in Animal Birthing^b^	Yes	186 (43.0)	69 (9.3)	< 0.001
	No	247 (57.0)	670 (90.7)	
Handle Raw Hides	Yes	172 (30.6)	54 (5.5)	< 0.001
	No	390 (69.4)	928 (94.5)	
Clean Barns	Yes	416 (74.0)	635 (64.7)	< 0.001
	No	146 (26.0)	347 (35.3)	

^a^Includes participants from households that practiced either pastoralism or mixed farming

^b^Variable has missing values

Apart from routine livestock husbandry practices such as feeding and cleaning animal barns, we compared cultural and occupational practices associated with *Brucella* spp. IgG seropositivity between enrolled household members from mixed farming and nomadic pastoralist households. The practices assessed were drinking unboiled milk, drinking raw blood, assisting animals during birth, and handling raw hides.

More than 79% of nomadic pastoralists consumed unboiled milk when compared to 1.7% of the mixed farmers (*p* < 0.001), and 28.3% of nomadic pastoralists consumed raw blood compared to 0.4% among mixed farmers (*p* < 0.001). In addition, up to 43% of nomadic pastoralists assisted in animal birth or handled raw hides when compared to less than10% among mixed farmers (*p* < 0.001) (Table 3).

### Association between practices and *Brucella* IgG sero-positivity

In the bivariate analyses, consuming raw blood or unboiled milk, assisting animals in birth, nomadic pastoralism, and handling livestock hides were significantly associated with brucellosis seropositivity (Table 4). Among participants who were seropositive for *Brucella spp.,* 68% reported consuming unboiled milk compared to 14.6% who drank unboiled milk and were seronegative (cOR[95% CI] = 12.5 [9.6–16.4]), whereas 24.6% reported consuming raw blood and were seropositive compared to 5.3% of those reported consuming raw blood and were seronegative (cOR[95%CI] = 5.8 [4.2–8.0]. Similarly, 42% of participants reported assisting in animal births and were seropositive compared to 17% who assisted in birthing but were seronegative (cOR[95%CI] = 3.6 [2.7–4.9] (Table 4). The multivariate mixed effects logistic regression model identified drinking unboiled milk and being from a household that practiced nomadic pastoralism as independent risk practices associated with *Brucella spp.* IgG seropositivity. The odds of brucellosis seropositivity were 8-fold higher among nomadic pastoralists compared to mixed farmers, (aOR[95%CI] = 8.6 [3.6–20.2]), after adjusting for other practices and sociodemographic characteristics (Table 4). Participants having formal education was a protective factor against *Brucella* spp. seropositivity.

**Table 4:** Bivariable and multivariable mixed effects logistic regression for the association between participant demographic characteristics and practices and *Brucella* spp. seropositivity, 2012–2013

Variable	Category	*Brucella* spp. seropositivity		Bivariable analysis	Multivariable mixed effects logistic model
		Positive	Negative	Crude OR (95% CI)	Adjusted OR (95% CI)
Sex	Male	191 (53.5)	719 (43.7)	1.5 (1.2–1.9)	1.46 (1.0–2.2)
	Female	166 (46.5)	928 (56.3)	ref^β^	ref^β^
Age, years	Mean (SD)	37.8 (19.9)	35.3 (19.4)	1.0 (1.0–1.0)	1.0 (1.0–1.0)
Highest education level completed	Primary	75 (21.1)	682 (41.6)	0.1 (0.1–0.2)	0.5 (0.3–1.0)
	Secondary	16 (4.5)	489 (29.8)	< 0.1 (< 0.1–0.1)	0.2 (0.1–0.6)
	Post secondary	4 (1.12)	163 (9.9)	< 0.1 (< 0.1–0.1)	0.4 (0.1–1.5)
	No Formal Education	261 (73.3)	306 (18.7)	ref^β^	ref^β^
Production system	Nomadic Pastoralist	272 (88.3)	286 (23.3)	24.7 (17.2–36.3)	8.6 (3.6–20.2)
	Mixed Farming	36 (11.7)	939 (76.7)	ref^β^	ref^β^
Drink unboiled milk	Yes	227 (68.2)	233 (14.6)	12.5 (9.6–16.4)	2.8 (1.4–5.3)
	No	106 (31.8)	1364 (85.4)	ref^β^	ref^β^
Drink raw blood	Yes	88 (24.6)	88 (5.3)	5.8 (4.2–8.0)	1.1 (0.6–1.8)
	No	269 (75.4)	1559 (94.7)	ref^β^	ref^β^
Assist in animal birthing	Yes	107 (42.1)	170 (16.7)	3.6 (2.7–4.9)	0.9 (0.5–1.4)
	No	147 (57.9)	845 (83.3)	ref^β^	ref^β^
Handle raw hides	Yes	97 (27.2)	148 (9.0)	3.8 (2.8–5.0)	1.0 (0.6–1.6)
	No	260 (72.8)	1499 (91.0)	ref^β^	ref^β^
Clean barns	Yes	240 (67.2)	909 (55.2)	1.7 (1.3–2.1)	1.0 (0.5–1.2)
	No	117 (32.8)	738 (44.8)	ref^β^	ref^β^

*ref*^*β*^ Reference category

## Discussion

An important finding in this study was that whereas over 70% of both nomadic pastoralists and mixed farmers had heard of brucellosis disease and had knowledge of common symptom (chills and loss of appetite), less than10% could identify key risky practices associated with brucellosis infection in humans including consuming raw dairy products and contact with aborted fetuses. Despite these comparable levels of knowledge of the disease among the two communities, over two-thirds of nomadic pastoralists engaged in risky practices including consumption of unboiled milk and raw blood, assisting with animal birth and handling raw hides. In contrast, less than 10% of mixed farmers engaged in these practices, including about 2% in consumption of unboiled milk or raw blood. Our findings show a strong link between these practices and *Brucella* spp. seropositivity, including a nearly 3-fold higher odds of seropositivity for people who consumed unboiled milk. These practices combined with the high brucellosis seroprevalence (13.5%) among their livestock, predisposes nomadic pastoralists to high *Brucella* spp. sero-positivity.

Our study found that two-thirds of the pastoralists had no formal education whereas almost all (92%) of the mixed farmers had at least primary school education, including almost half that had secondary school education or higher. The low level of formal education among pastoralists, who typically live in expansive and sparsely populated remote semi-arid and arid lands of sub-Saharan Africa, when compared to other communities living in more developed and agriculturally productive regions, is associated with underdevelopment and poor infrastructures, and the occupation and lifestyle of pastoralists [19–21]. Despite this disparity in education, our study found the two livestock-owning communities with comparable knowledge levels of brucellosis disease, including a moderate to high knowledge of its existence and its effects on humans and low knowledge such as mechanisms of animal-to-human transmission and prevention and control measures. This finding is in agreement with other studies showing there is significant knowledge of common endemic zoonotic disease including brucellosis, echinococcosis, and rabies exists among nomadic pastoralists despite the lack of formal education [22].

The question is why pastoralists engage in risky practices given their knowledge level is similar to mixed farmers. One possibility is that nomadic pastoralists, perhaps because of low levels of formal education and representation in national leadership, distrust the government health services, resulting in low receptiveness to public health and animal health education on disease prevention and control [22]. This is unlikely because our experience during field studies and vaccination campaigns show comparable reception of among all communities. A more plausible explanation is that lack of other sources of livelihood and occupations, apart from rearing livestock in these arid lands, leaves pastoralists with no choice but to engage in risky practices in the course of their interactions with livestock - perhaps even when they know the associated risks. Our interactions with nomadic pastoralists including some with education and knowledge of risk factors of brucellosis revealed that they engage in risky practices because of culture. Social studies to understand why people engage in risky practices such as commercial sex workers engaging in unsafe sex, drug users, and youth engaging in texting while driving identified economic insecurity and culture as possible reasons [23–26]. The studies among drug users involved in risky income generation showed that almost half of them would continue the risky practices even if they went off drugs but remained economically insecure [23]. The primary occupation of nomadic pastoralists is to herd livestock and use these and their products including milk, meat, fur, hides, leather and manure for their livelihood and socio-economic advancement. They routinely give extra care to pregnant livestock, which are typically kept near pasture areas to reduce the long treks that other animals undertake and including assisting them with birth and management of the newborn animals. In addition, they routinely drink raw blood and unboiled milk obtained for survival during their movement across the arid lands [9, 27, 28]. Nomadic pastoralists believe that unboiled camel milk has better taste and it possesses medicinal value including aphrodisiac properties [27–31]. In addition, they engaged in skinning and therefore handling raw hides because of the high market value of camel leather and hair [32]. Since our study also found that lack of formal education was an important risk factor associated with increased risk of brucellosis infection, we envision that promoting formal education among the pastoralists would result in improved economic opportunities and reduction in risky practices as has been observed in other studies [33, 34].

Previous findings showed over 65% household prevalence of brucellosis among the nomadic pastoralist communities of Marsabit County, which was 12-fold higher than other regions of the country [10]. The findings of our current study suggest that health education should emphasize risky cultural practices and accompanied by increased formal education and economic renaissance. It is likely the recent introduction of a devolved government in Kenya, which increased public participation and resource allocation to such marginalized areas will begin to turn the tide of such endemic zoonotic diseases. However, a more effective approach should be to promote formal education and development and implement a prevention and control strategy targeting reduction of the disease in both livestock through routine vaccination, and public education among humans to curb the risky practices we identified.

This study had some limitations. First, the exclusion of children below 5 years of age limits the generalizability of our data to the entire population. Our determination of risky practices was based on interviews, which could likely introduce information bias with participants giving responses they consider favorable. We think that this possible bias was minimal based on findings of the key informant interviews on the prevalence of the practices in the communities.

The little knowledge of how brucellosis is transmitted to humans and among animals suggest that more public education on the disease would be useful, while at the same time developing behavior change communication strategies for different communities is critical. In addition, there is need for collaboration between the veterinary and public health professionals through the one health approach in the provision of health education and information including symptoms, transmission pathways and prevention of brucellosis at community level to reduce disease prevalence.

## Conclusions

Our findings show that despite comparable levels of knowledge of brucellosis disease by both nomadic pastoralists and mixed farmers, over two-thirds of nomadic pastoralists engaged in at least one of four key risky practices including consumption of unboiled milk and raw blood, assisting with animal birth and handling raw hides. We also found a strong link between these practices and *Brucella* spp. seropositivity, including a nearly 3-fold higher odds of seropositivity for people who consumed unboiled milk. We argue that nomadic pastoralists are likely to engage in risky practices which promote *Brucella spp.* infection probably related to occupation and culture, despite having significant knowledge of the disease.

## Supplementary information

**Additional file 1.** STROBE Checklist.

## Data Availability

The questionnaire, datasets used and/or analysed during the current study are available from the corresponding author on reasonable request.

## Abbreviations

aOR: Adjusted Odds Ratio
CI: Confidence Interval
cOR: Crude Odds Ratio
IgG: Immunoglobulin G
